# Breastfeeding in relation to risk of different breast cancer characteristics

**DOI:** 10.1186/1756-0500-7-216

**Published:** 2014-04-07

**Authors:** Salma Butt, Signe Borgquist, Lola Anagnostaki, Göran Landberg, Jonas Manjer

**Affiliations:** 1 Department of Surgery, Skåne University Hospital, Malmö, Lund University, Malmö, Sweden; 2 Malmö Diet and Cancer Study, Skåne University Hospital, Malmö, Sweden; 3 Centre for Molecular Pathology, Department of Laboratory Medicine, Skåne University Hospital, Malmö, Sweden; 4 Division of Oncology, Department of Clinical Sciences, Skåne University Hospital, Lund, Sweden

**Keywords:** Breast cancer risk, Breastfeeding, Stage, Characteristics

## Abstract

**Background:**

The aim of this present study was to examine duration of breastfeeding in relation to the risk of different subgroups of breast cancer. A prospective cohort, The Malmö Diet and Cancer study, including 14092 parous women, were followed during a mean of 10.2 years and a total of 424 incident breast cancers were diagnosed.

**Methods:**

Tumours were classified regarding invasiveness, tumour size, axillary lymph node status, Nottingham grade, tumour proliferation (Ki67), HER2, cyclin D1 and p27, WHO histological type and hormone receptor status. Duration of breastfeeding was measured using total time of breastfeeding, categorized in quartiles using the lowest as the reference group (<4.0, ≥4.0- < 8.0, ≥8.0- < 13.0 and ≥13.0 months). Average duration of breastfeeding per child and breastfeeding duration of the first child were also used as exposures in separate analyses. Relative risks, with 95% confidence intervals, were obtained using a Cox’s proportional hazards analysis adjusted for potential confounders.

**Results:**

Overall risk for breast cancer was similar in all quartiles of breastfeeding. No strong results regarding breastfeeding duration and breast cancer subgroups were seen. A few results indicated an association between a relatively long duration of breastfeeding and tumours with high proliferation (Ki67) and grade III histological grade.

**Conclusions:**

Breastfeeding duration was not associated with breast cancer risk and no strong results were seen with regard to breast cancer subgroups.

## Background

Several early studies and a recently a large meta-analysis including data from thirty countries, have shown a negative association between increasing time of breastfeeding and the risk of breast cancer [1]. However, a number of studies have not been able to shown this association as reviewed by Yang et al. [2]. One reason for these inconclusive findings may be that breastfeeding only influences the risk of certain sub-groups of breast cancer. There has been a limited number of studies investigating breastfeeding in relation to different breast cancer sub-groups, and only characteristics such as histological type and estrogen receptor status have been investigated [3-5].

In all, 17 035 women participated in a prospective population-based cohort in Malmö, Sweden: The Malmö Diet and Cancer Study (MDCS). The MDCS collected information on breastfeeding, as well as other reproductive and environmental factors. During follow-up, 622 women were diagnosed with breast cancer. Tumour tissue samples were available for about 90% of these women, which allowed tumour reclassification and further tumour biological examinations. Tumours were evaluated with regard to invasiveness, size, axillary lymph node status, Nottingham grade, tumour proliferation (Ki67), Human epidermal growth factor receptor 2 (HER2) status, and expression of cell cycle regulators such as cyclin D1 and p27. Tumours were further examined for WHO histological type (ductal, lobular, and tubular) and hormone receptor status; estrogen receptor alpha (ERα), estrogen receptor beta (ERβ) and progesterone receptor (PgR).

The aim of the present study was to examine breastfeeding in relation to the risk of breast tumours with different biological characteristics.

## Methods

### The Malmö Diet and Cancer Study (The MDCS)

All women born between 1923 and 1950 in Malmö were invited to a prospective cohort study, the MDCS. Between the years 1991 and 1996, 17 035 women participated [6]. Written informed consent was obtained from all participants.

Information on breastfeeding, education, occupation, marital status, age at menarche, parity, year of each child’s birth, age at menopause, exposure to oral contraceptives (OC) (ever/never), current use of hormonal replacement therapy (HRT), alcohol consumption and smoking habits were collected using a questionnaire at baseline [7].

Menopausal status was assessed using both medical records and the questionnaire. A women was considered postmenopausal: (1) if she had undergone bilateral oophorectomy; or (2) if she had undergone hysterectomy but not bilateral oophorectomy, and if she was ≥55 years of age; or (3) if the above criteria were absent and she affirmed that her menstruations had ceased at least during the calendar year two years prior to baseline examination; or (4) if it was unknown whether or not she had undergone a previous oophorectomy or hysterectomy and information on menstrual status was missing, and she was ≥55 years of age. In all 11388 women were postmenopausal at baseline. A woman was classified as pre-/perimenopausal if she affirmed that she was still menstruating, or if her menstruations had ceased less than two years prior to baseline examinations, or if information on menstrual status was missing and the women was <55 years of age at baseline.

Height and weight was measured at baseline by a trained nurse at the study centre, and Body Mass Index (BMI) was calculated as kg/m^2^.

The MDCS and the present analyses were approved by the Ethical Committee at Lund University (LU 51–90 and Dnr 652/2005).

### Breastfeeding and parity

Time of breastfeeding was assessed with the help of a questionnaire. All participants were asked to fill in the number of children they had given birth to. Information on birth years of the children and duration of breastfeeding was retrieved for the first seven children. No information on twin pregnancies was available. Parity was defined as the total number of children that a women answered she had given birth to.

Mean duration of breastfeeding per child was calculated as the sum of the months of breastfeeding divided by the number of children with information on breastfeeding.

Total time of breastfeeding was calculated as mean time of breastfeeding multiplied with parity. This calculation was made since time of breastfeeding was limited to seven children and a small amount of women had eight or more children (n = 13). Breastfeeding of the first child was investigated in an additional analysis, as it is likely that changes during the first pregnancy and following lactation period may be particularly important with regard to the differentiation of the breast tissue [8]. Women that had never breastfed were included in the lowest quartile in all breastfeeding groups.

### Follow-up

All women were followed until 31 December 2004 with tumour end-points retrieved by record linkage with The Swedish Cancer Registry (until 31 December 2003). Due to a delay in central registration, linkage to its regional branch, The Southern Swedish Regional Tumour Registry, provided tumour-endpoints for the year 2004. Vital status was obtained from The Swedish Cause-of-Death Registry until 31 December 2004. A total of 622 incident breast cancer cases (including invasive and cancer in situ) were registered during follow-up.

### Study population

**Figure 1:**
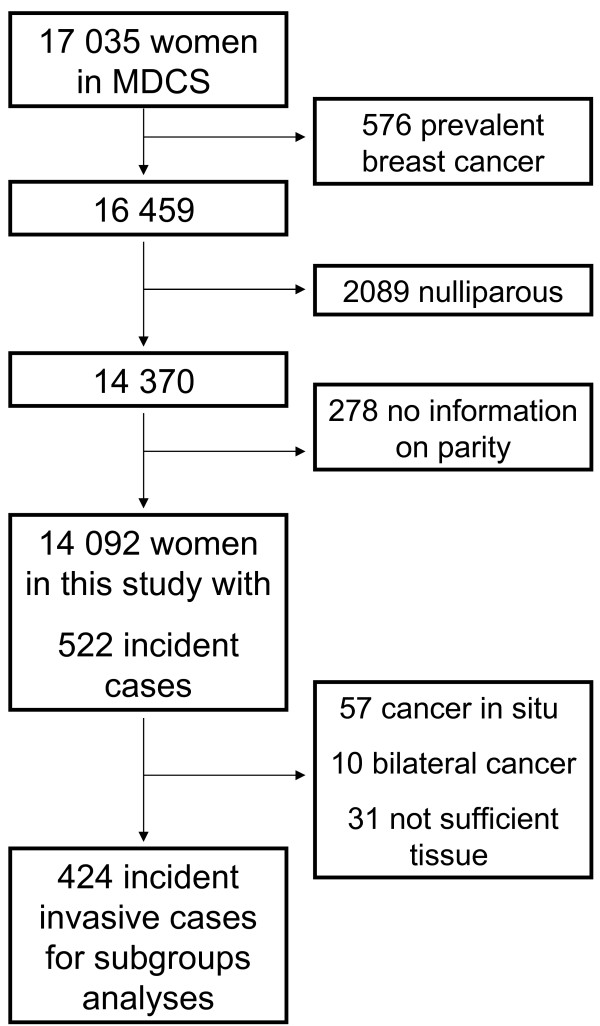
Study population (MDCS).

Out of 17 035 women, 576 had been diagnosed with breast cancer prior to inclusion in the study, and they were categorized as prevalent breast cancer cases and were subsequently excluded from the analyses. A total of 2089 women were nulliparous and hence excluded from the study population. Yet another 278 had no information on parity and were also excluded. This gave us a study population of 14 092 women with 522 incident breast cancer cases. A total of 57, were cancer in situ (CIS) and only provided person-years until event, but did not provide invasive end-points in the analyses for “all breast cancers”. CIS cases were neither included in the analyses of specific subgroups, e.g. histology and receptor status. Ten women with bilateral breast cancer were excluded as tumour end-points in the analyses due to difficulties in determining the relevant side to be used in the analyses of tumour size, axillary lymph nodes, histopathology and receptor status. A further 31 did not have sufficient tissue for further analyses. Bilateral cases and cases with no tumour material did however provide person-years up until the event. In all 424 tumours were included in the subgroup analyses (Figure 1).

### Histopathological analyses

One senior breast pathologist re-evaluated all invasive tumours (LA). All tumours were re-evaluated concerning invasiveness and tumour type was described according to the WHO classification. The tumours were graded according to Elston and Ellis, including tubular formation, nuclear atypia and mitotic index [9]. Further tumour characteristics were evaluated using the tissue microarray technique (TMA). For the construction of tissue micro array, described elsewhere [10,11] two cores of 0.6 mm from each tumour were taken and arranged in a recipient block. Immunohistochemical (IHC) analyses were performed using specific antibodies, described previously by Borgquist et al. [10] and tumours were evaluated according to the nucleus expression of ERα, ERβ, PgR, Ki67, cyclin D1 and p27. Tumours were dichotomized as negative and positive using the categories 0–10% and 11-100% of positive nuclei respectively. HER2 was analyzed using IHC as previously described [10]. HER2 was classified according to the Swedish clinical practice [12]. As data on HER2 amplification was not available, HER2 status was based on IHC analyses and in this study dichotomized into negative (0 and 1+) or positive cases (2+ and 3+). All arrays were evaluated independently twice by the same person (SiB) and in case of discrepancy, a third evaluation was performed by the same investigator.

Information on tumour laterality, size and lymph node metastasis were retrieved from medical records and histopathological reports by one registered nurse.

### Statistical methods

The main analyses used total duration of breastfeeding as measurements. Additional analyses used mean duration of breastfeeding and breastfeeding duration of the first child. Breastfeeding duration was divided into quartiles. Quartile cut-offs for breastfeeding were based on the distribution of all women in the study cohort. Different quartiles of breastfeeding were compared regarding the distribution of established and potential risk factors for breast cancer. Each subject was followed until the event of breast cancer, death or end of follow- up, 31 December 2004. The incidence of breast cancer was calculated per 100,000 person-years in different breastfeeding quartiles. Corresponding relative risks of breast cancer were analyzed using a Cox’s proportional hazards analysis yielding relative risks (RR) with 95% confidence intervals (CI). These analyses were subsequently adjusted for potential confounders; age at baseline, education, socioeconomic status, marital status, age at menarche, age at first birth, parity, oophorectomy, age at menopause, oral contraceptive use, hormone replacement therapy use, bmi, alcohol consumption, smoking and height (categorized in Table 1).

**Table 1:** Distribution of risk factors in different quartiles of total duration of breastfeeding

**Time in months:**	**<4.0**	**≥4.0 - <8.0**	**≥8.0 - <13.0**	**≥13.0**	**Missing**
**Total: 14092**	**n = 2908**	**n = 3688**	**n = 3484**	**n = 3451**	**n = 561**
	**Column percent** ***(mean and SD in italics)***				
Age at baseline (years)					
*Mean (SD)*	*55.9 (7.7)*	*56.7 (7.8)*	*57.6 (7.7)*	*58.5 (8.1)*	*57.7 (6.7)*
Education (n)					
O-level college	74.0	72.7	69.0	65.6	80.6
A-level college	7.7	6.7	7.4	6.2	5.0
University	18.3	20.4	23.2	28.0	13.0
Type of occupation					
Manual worker	40.7	39.3	37.9	40.5	45.6
Non-manual worker	51.0	52.9	53.6	49.7	47.1
Employer-self-employed	7.3	7.1	7.5	8.3	5.5
Married/cohabiting					
No	32.5	30.2	27.5	28.8	35.5
Yes	67.5	69.8	72.5	71.2	64.2
Age at menarche					
≤12	23.6	21.3	21.6	22.2	22.5
>12 to <15	53.2	54.8	52.9	51.8	47.1
≥15	22.7	23.2	25.1	25.5	27.1
Parity					
1	54.9	30.5	14.1	1.2	40.1
2	35.1	55.7	59.5	41.6	36.5
≥3	10.0	13.9	26.4	57.2	23.4
Age at first childbirth					
≤20	17.0	19.7	18.8	22.3	19.4
>20 to ≤25	38.1	41.6	42.1	43.1	34.9
>25 to ≤30	29.7	28.2	29.4	26.5	28.3
>30	15.1	10.4	9.7	8.1	15.9
Bilateral oophorectomy					
No	98.7	98.4	98.6	98.7	98.8
Yes	1.3	1.6	1.4	1.3	1.2
Age at menopause					
Pre-/perimenopausal	39.3	36.5	31.4	30.0	26.0
≤45	14.5	12.2	11.6	11.3	15.0
>45 to <53	34.7	36.4	40.0	39.9	43.1
≥53	10.2	13.1	15.6	17.4	12.5
Exposure to OC (ever/never)					
No	47.3	45.7	49.3	53.5	52.8
Yes	52.6	54.2	50.7	46.5	46.7
Exposure to HRT*					
No	79.2	79.7	79.9	82.8	81.7
ERT	8.0	7.5	8.4	7.8	6.0
PRT	0.5	0.3	0.2	0.3	0.0
CHRT	12.0	12.0	11.1	8.8	12.1
Height					
*Mean (SD)*	*163.5 (6.0)*	*163.8 (6.0)*	*163.6 (5.9)*	*163.4 (6.1)*	*162.8 (6.2)*
Body mass index					
*Mean (SD)*	*25.5 (4.4)*	*25.3 (4.5)*	*25.3 (4.2)*	*25.7 (4.4)*	*26.0 (4.8)*
Alcohol consumption					
Nothing last year (teetotaler)	10.9	9.2	10.3	13.8	17.5
Something last year (not last month)	12.1	11.4	12.0	12.2	13.7
Something last month	76.8	79.3	77.6	73.8	67.6
Smoking					
Never	38.2	40.6	45.4	49.9	44.4
Current	33.2	30.6	26.5	23.0	33.9
Ex	28.5	28.8	28.1	27.1	21.7

Percentages do not always add up to 100% due to missing. *Current in peri- and postmenopausal women n = 10288.

Trend over breastfeeding categories was examined from the lowest to the highest quartile, excluding the missing category.

To examine heterogeneity, to test whether effect estimates were similar between for example grade I and grade III tumours in a certain breastfeeding quartile, adjusted case-case models using unconditional logistic regression analysis were used and p-values <0.05 were considered statistically significant.

## Results

### Total duration of breastfeeding in relation to risk factors for breast cancer

Women in the highest quartile of breastfeeding duration were more often multiparous and younger at first childbirth, as compared to all other groups. Moreover, women in the highest quartile were older at menopause, were less exposed to oral contraceptives and had to a higher extent never smoked (Table 1). All other factors were evenly distributed between breastfeeding categories.

### Total duration of breastfeeding in relation to risk of different breast cancer subgroups

The overall risk of breast cancer (i.e. the risk of unilateral invasive tumours with biological material) was similar in all breastfeeding groups as compared to the lower quartile (Table 2). There was a trend towards grade III tumours in women with higher duration of breastfeeding, however this association did not reach statistical significance (Table 2). The risk of high Ki67 expressing tumours was statistically significantly associated with increased duration of breastfeeding (Table 3). Women in the lowest breastfeeding quartile had a higher risk of expressing ductal type of breast tumour, however this results was not statistically significant (Table 4).

**Table 2:** Risk of breast cancer subgroups defined by clinico-pathological markers in relation to total duration of breastfeeding

**Tumour subgroup**	**Breastfeeding total in months**	**Number of cases**	**Incidence/100000**	**RR**	RR*
Invasive					
breast cancer**	<4.0	80	270	1.00	1.00
	≥4.0 - <8.0	108	288	1.07 (0.80 - 1.42)	1.04 (0.77 - 1.40)
	≥8.0 - <13.0	109	304	1.12 (0.84 - 1.49)	1.09 (0.80 - 1.48)
	≥13.0	103	293	1.08 (0.81 - 1.45)	1.10 (0.78 - 1.54)
	Missing	24	399	---	---
	Total	424	294	*p-trend: 0.56*	*p-trend:0.45*
CIS					
	<4.0	9	30	1.00	1.00
	≥4.0 - <8.0	19	51	1.67 (0.76 - 3.70)	1.90 (0.84 - 4.30)
	≥8.0 - <13.0	13	36	1.20 (0.51 - 2.81)	1.40 (0.57 - 3.46)
	≥13.0	13	37	1.22 (0.52 - 2.86)	1.59 (0.60 - 4.21)
	Missing	3	50	---	---
	Total	57	40	*p-trend:0.98*	*p-trend:0.48*
Size ≤20 mm^#^					
	<4.0	55	186	1. 00	1.00
	≥4.0 - <8.0	80	214	1.15 (0.82 - 1.62)	1.12 (0.79 - 1.60)
	≥8.0 - <13.0	69	192	1.03 (0.72 - 1.47)	0.99 (0.68 - 1.45)
	≥13.0	85	241	1.30 (0.93 - 1.82)	1.29 (0.87 - 1.92)
	Missing	15	249	---	---
	Total	304	211	*p-trend:0.21*	*p-trend:0.24*
Size >20 mm					
	<4.0	25	84	1.00	1.00
	≥4.0 - <8.0	27	72	0.85 (0.50 - 1.47)	0.84 (0.48 - 1.47)
	≥8.0 - <13.0	39	109	1.28 (0.78 - 2.12)	1.28 (0.75 - 2.20)
	≥13.0	18	51	0.61 (0.33 - 1.11)	0.65 (0.33 - 1.27)
	Missing	9	150	---	---
	Total	118	82	*p-trend:0.36*	*p-trend:0.63*
Axillary lymph					
node neg^#^	<4.0	27	91	1.00	1.00
	≥4.0 - <8.0	32	85	0.94 (0.56 - 1.56)	0.91 (0.54 - 1.54)
	≥8.0 - <13.0	34	95	1.04 (0.63 - 1.72)	0.99 (0.56 - 1.70)
	≥13.0	31	88	0.97 (0.58 - 1.62)	0.90 (0.50 - 1.64)
	Missing	5	83	---	---
	Total	129	89	*p-trend:0.99*	*p-trend:0.82*
Axillary lymph					
node pos	<4.0	53	179	1.00	1.00
	≥4.0 - <8.0	72	192	1.07 (0.75 - 1.53)	1.05 (0.73 - 1.51)
	≥8.0 - <13.0	71	198	1.10 (0.77 - 1.57)	1.08 (0.73 - 1.57)
	≥13.0	71	202	1.13 (0.79 - 1.61)	1.18 (0.78 - 1.79)
	Missing	19	316	---	---
	Total	286	198	*p-trend: 0.52*	*p-trend:0.30*
Grade I^#^					
	<4.0	27	91	1.00	1.00
	≥4.0 - <8.0	37	99	1.08 (0.66 - 1.78)	1.01 (0.61 - 1.69)
	≥8.0 - <13.0	28	78	0.85 (0.50 - 1.45)	0.78 (0.44 - 1.38)
	≥13.0	28	80	0.87 (0.51 - 1.48)	0.87 (0.47 - 1.61)
	Missing	7	116	---	---
	Total	127	88	*p-trend:0.42*	*p-trend:0.71*
Grade II					
	<4.0	33	111	1.00	1.00
	≥4.0 - <8.0	47	125	1.12 (0.72 - 1.75)	1.05 (0.67 - 1.66)
	≥8.0 - <13.0	53	148	1.32 (0.85 - 2.03)	1.15 (0.73 - 1.83)
	≥13.0	46	131	1.17 (0.75 - 1.83)	0.99 (0.60 - 1.64)
	Missing	12	199	---	---
	Total	191	132	*p-trend:0.40*	*p-trend:0.97*
Grade III^##^					
	<4.0	20	67	1.00	1.00
	≥4.0 - <8.0	23	61	0.91 (0.45 - 1.66)	1.00 (0.54 - 1.85)
	≥8.0 - <13.0	28	78	1.15 (0.65 - 2.05)	1.43 (0.77 - 2.66)
	≥13.0	29	82	1.22 (0.69 - 2.16)	1.74 (0.89 - 3.41)
	Missing	5	83	---	---
	Total	105	73	*p-trend:0.34*	*p-trend:0.051*

*Adjusted for age at baseline (continuous), education, socioeconomic status, marital status, age at menarche, age at first birth, parity, oophorectomy, age at menopause, oral contraceptive use, hormone replacement therapy use, bmi, alcohol consumption, smoking and height.

**All cases with unilateral invasive breast cancer with tissue samples available for examination.

^#^Reference group in heterogeneity analyses. ^##^Heterogeneity: p = 0.031.

**Table 3:** Risk of breast cancer subgroups defined by immunohistochemical markers in relation to total duration of breastfeeding

**Tumour subgroup**	**Breastfeeding total in months**	**Number of cases**	**Incidence/100000**	**RR**	RR*
Ki67 low					
(≤10%)^#^	<4.0	54	182	1.00	1.00
	≥4.0 - <8.0	76	203	1.11 (0.78 - 1.57)	1.06 (0.74 - 1.51)
	≥8.0 - <13.0	72	201	1.09 (0.77 - 1.56)	1.01 (0.69 - 1.47)
	≥13.0	58	165	0.90 (0.62 - 1.31)	0.82 (0.53 - 1.25)
	Missing	11	183	---	---
	Total	271	188	*p-trend:0.54*	*p-trend:0.44*
Ki67 high					
(>10%)	<4.0	17	57	1.00	1.00
	≥4.0 - <8.0	21	56	0.98 (0.51 - 1.85)	1.02 (0.53 - 1.96)
	≥8.0 - <13.0	26	72	1.25 (0.68 - 2.31)	1.39 (0.72 - 2.67)
	≥13.0	30	85	1.48 (0.82 - 2.69)	1.88 (0.94 - 3.76)
	Missing	7	116	---	---
	Total	101	70	*p-trend:0.11*	*p-trend:0.03*
HER2 (0-1+)^#^					
	<4.0	57	192	1.00	1.00
	≥4.0 - <8.0	78	208	1.08 (0.77 - 1.52)	1.02 (0.72 - 1.45)
	≥8.0 - <13.0	84	234	1.21 (0.86 - 1.69)	1.12 (0.78 - 1.61)
	≥13.0	73	207	1.07 (0.76 - 1.52)	1.07 (0.72 - 1.59)
	Missing	19	499	---	---
	Total	311	216	*p-trend:0.58*	*p-trend:0.44*
HER2 (2 + -3+)					
	<4.0	9	30	1.00	1.00
	≥4.0 - <8.0	9	24	0.80 (0.32 - 2.01)	1.02 (0.39 - 2.65)
	≥8.0 - <13.0	11	31	1.02 (0.42 - 2.46)	1.40 (0.53 - 3.69)
	≥13.0	11	31	1.03 (0.43 - 2.49)	1.35 (0.46 - 3.97)
	Missing	1	17	---	---
	Total	41	28	*p-trend:0.79*	*p-trend:0.59*
Cyclin D1 low					
(≤10%) ^#^	<4.0	54	182	1.00	1.00
	≥4.0 - <8.0	82	219	1.20 (0.85 - 1.69)	1.15 (0.81 - 1.64)
	≥8.0 - <13.0	73	203	1.11 (0.78 - 1.58)	1.05 (0.72 - 1.53)
	≥13.0	73	122	1.14 (0.80 - 1.62)	1.06 (0.71 - 1.60)
	Missing	13	216	---	---
	Total	295	205	*p-trend:0.65*	*p-trend:0.77*
Cyclin D1 high					
(>10%)	<4.0	14	47	1.00	1.00
	≥4.0 - <8.0	14	37	0.79 (0.37 - 1.65)	0.84 (0.39 - 1.78)
	≥8.0 - <13.0	24	67	1.38 (0.72 - 2.68)	1.56 (0.77 - 3.19)
	≥13.0	20	57	1.19 (0.60 - 2.36)	1.61 (0.73 - 3.56)
	Missing	6	100	---	---
	Total	78	54	*p-trend:0.29*	*p-trend:0.10*
P27 low					
(≤10%) ^#^	<4.0	21	71	1.00	1.00
	≥4.0 - <8.0	41	109	1.55 (0.91 - 2.62)	1.56 (0.91 - 2.67)
	≥8.0 - <13.0	31	86	1.22 (0.70 - 2.12)	1.21 (0.68 - 2.18)
	≥13.0	35	99	1.40 (0.82 - 2.41)	1.35 (0.73 - 2.50)
	Missing	7	116	---	---
	Total	135	94	*p-trend:0.47*	*p-trend:0.61*
P27 high					
(>10%)	<4.0	48	162	1.00	1.00
	≥4.0 - <8.0	53	141	0.87 (0.59 - 1.29)	0.83 (0.55 - 1.24)
	≥8.0 - <13.0	65	181	1.11 (0.76 - 1.61)	1.04 (0.70 - 1.56)
	≥13.0	53	151	0.93 (0.63 - 1.37)	0.93 (0.59 - 1.46)
	Missing	11	183	---	---
	Total	230	159	*p-trend:0.95*	*p-trend:0.73*

*Adjusted for age at baseline (continuous), education, socioeconomic status, marital status, age at menarche, age at first birth, parity, oophorectomy, age at menopause, oral contraceptive use, hormone replacement therapy use, bmi, alcohol consumption, smoking and height.

^#^Reference group in heterogeneity analyses.

**Table 4:** Risk of breast cancer subgroups defined by type and receptor status in relation to total duration of breastfeeding

**Tumour subgroup**	**Breastfeeding total in months**	**Number of cases**	**Incidence/100000**	**RR**	RR*
Ductal^#^					
	<4.0	59	199	1.00	1.00
	≥4.0 - <8.0	74	197	0.99 (0.70 - 1.39)	0.99 (0.70 - 1.41)
	≥8.0 - <13.0	74	206	1.03 (0.73 - 1.45)	1.04 (0.72 - 1.51)
	≥13.0	79	224	1.12 (0.80 - 1.58)	1.20 (0.81 - 1.78)
	Missing	12	199	---	---
	Total	298	207	*p-trend:0.45*	*p-trend:0.26*
Lobular					
	<4.0	15	51	1.00	1.00
	≥4.0 - <8.0	21	56	1.11 (0.57 - 2.15)	0.95 (0.48 - 1.88)
	≥8.0 - <13.0	23	64	1.27 (0.66 - 2.43)	1.02 (0.51 - 2.04)
	≥13.0	14	40	0.79 (0.38 - 1.63)	0.65 (0.29 - 1.45)
	Missing	9	150	---	---
	Total	82	57	*p-trend:0.63*	*p-trend:0.44*
Tubular					
	<4.0	4	13	1.00	1.00
	≥4.0 - <8.0	10	27	1.97 (0.62 - 6.29)	2.09 (0.64 - 6.89)
	≥8.0 - <13.0	7	20	1.43 (0.42 - 4.89)	1.59 (0.43 - 5.91)
	≥13.0	6	17	1.26 (0.36 - 4.47)	1.56 (0.37 - 6.70)
	Missing	2	33	---	---
	Total	29	20	*p-trend:.1.00*	*p-trend:0.75*
ERα neg ≤10%^#^					
	<4.0	9	30	1.00	1.00
	≥4.0 - <8.0	12	32	1.05 (0.44 - 2.50)	1.16 (0.48 - 2.83)
	≥8.0 - <13.0	13	36	1.18 (0.50 - 2.75)	1.38 (0.55 - 3.47)
	≥13.0	15	43	1.40 (0.61 - 3.19)	1.61 (0.60 - 4.35)
	Missing	1	17	---	---
	Total	50	35	*p-trend:0.38*	*p-trend:0.30*
ERα pos >10%					
	<4.0	61	206	1.00	1.00
	≥4.0 - <8.0	85	227	1.10 (0.79 - 1.53)	1.06 (0.75 - 1.48)
	≥8.0 - <13.0	88	245	1.19 (0.86 - 1.64)	1.13 (0.79 - 1.60)
	≥13.0	80	227	1.10 (0.79 - 1.54)	1.09 (0.75 - 1.61)
	Missing	20	332	---	---
	Total	334	232	*p-trend:0.52*	*p-trend:0.47*
ERβ neg ≤10%^#^					
	<4.0	29	98	1.00	1.00
	≥4.0 - <8.0	38	101	1.04 (0.64 - 1.68)	1.04 (0.63 - 1.70)
	≥8.0 - <13.0	44	123	1.26 (0.79 - 2.01)	1.26 (0.76 - 2.09)
	≥13.0	40	114	1.16 (0.72 - 1.87)	1.20 (0.69 - 2.09)
	Missing	9	150	---	---
	Total	160	111	*p-trend:0.40*	*p-trend:0.32*
ERβ pos >10%					
	<4.0	33	111	1.00	1.00
	≥4.0 - <8.0	41	109	0.98 (0.62 - 1.54)	0.95 (0.59 - 1.53)
	≥8.0 - <13.0	40	111	0.98 (0.62 - 1.55)	0.93 (0.57 - 1.53)
	≥13.0	33	94	0.84 (0.52 - 1.35)	0.80 (0.46 - 1.40)
	Missing	9	150	---	---
	Total	156	108	*p-trend:0.48*	*p-trend:0.62*
PgR neg ≤10%^#^					
	<4.0	42	142	1.00	1.00
	≥4.0 - <8.0	49	131	0.92 (0.61 - 1.39)	0.91 (0.59 - 1.38)
	≥8.0 - <13.0	50	139	0.98 (0.65 - 1.48)	0.96 (0.62 - 1.49)
	≥13.0	49	139	0.98 (0.65 - 1.48)	1.01 (0.63 - 1.63)
	Missing	9	150	---	---
	Total	199	138	*p-trend:0.98*	*p-trend:0.90*
PgR pos >10%					
	<4.0	28	94	1.00	1.00
	≥4.0 - <8.0	40	107	1.12 (0.69 - 1.82)	1.13 (0.69 - 1.86)
	≥8.0 - <13.0	43	120	1.25 (0.78 - 2.02)	1.30 (0.78 - 2.16)
	≥13.0	38	108	1.14 (0.70 - 1.85)	1.24 (0.70 - 2.17)
	Missing	10	166	---	---
	Total	159	110	*p-trend:0.55*	*p-trend:0.29*

*Adjusted for age at baseline (continuous), education, socioeconomic status, marital status, age at menarche, age at first birth, parity, oophorectomy, age at menopause, oral contraceptive use, hormone replacement therapy use, bmi, alcohol consumption, smoking and height.

^#^Reference group in heterogeneity analyses.

### Average duration of breastfeeding

Women in the lowest quartile of average duration of breastfeeding were younger at baseline and were more often pre-/perimenopausal at breast cancer diagnosis as compared to all other groups (Additional file 1: Table S1). The relative risks for average duration of breastfeeding were similar to relative risks of total duration of breastfeeding. The risk of having grade III tumours were statistically significant for women in the highest quartile and the trend for having grade III tumours with increasing time of breastfeeding duration reached statistical significance (Additional file 1: Table S2). Moreover, no higher risk for tubular type was seen in any breastfeeding category (Additional file 1: Table S4).

### Breastfeeding duration of first child

The analyses of breastfeeding of first child in relation to risk factors for breast cancer showed that women in the highest quartile were more likely to have had at later first childbirth as compared to the other breastfeeding groups. All other risk factors were distributed as for total duration of breastfeeding (Additional file 1: Table S5). All relative risks for breastfeeding of the first childbirth in relation to risk of different breast cancer subgroups were similar to those related to total duration of breastfeeding and average duration of breastfeeding (Additional file 1: Table S6-S8). There was a statistically significant increased risk of having high expression of cyclin D1 with increasing duration of breastfeeding (Additional file 1: Table S7).

## Discussion

The aim of this study was to investigate breastfeeding in relation to the risk of different breast cancer subtypes and could not find any strong results. There was a trend towards more grade III tumours and high Ki67 expression with increasing duration of breastfeeding. However a lot of markers were tested and the quartile risks were not statistically significant for grade and Ki67 when studying total duration of breastfeeding.

### Methodological considerations

Information on breastfeeding was retrieved from a questionnaire provided at baseline examinations where all women were 44 years or older, thus unlikely to have additional children following baseline. A previous study has confirmed self-reported breastfeeding to be highly accurate [13], hence we consider this information to be valid. A limitation of this study is that the questionnaire did not allow for a distinction between different breastfeeding patterns. That is, some women may have reported the time they were exclusively breastfeeding, whilst others may have filled in total duration of breastfeeding. There may also be secular trend in breastfeeding patterns, as the recommendations for exclusive/partial breastfeeding have changed over time in Sweden [14]. Today, Sweden is a country with high rate of women breastfeeding for at least six months [14], yielding a study population that is most likely affected by these patterns. There is still no established “best way” of defining the amount of breastfeeding. Most previous studies have used ever/never breastfeeding as exposure. In the present study, there were 680 women (4.8%) who reported that they had never breastfed. When the present data was re-analyzed using this categorization, there was no statistically significant association between overall breast cancer risk and ever breastfeeding (adjusted relative risk =1.14: 0.70-1.88) as compared to never. Indeed, we consider it more valuable to investigate total time of breastfeeding in relation to different breast cancer characteristics in order to see if there was a threshold.

All tumour endpoints were retrieved by record linkage to The Swedish Cancer Registry. This is a nation-wide registry and all cancer cases in Sweden are to be reported to this registry. This registry has previously been validated in Malmö and the completeness was 99% regarding breast cancer [15].

Women with prevalent breast cancer were not included in the analyses since these women most likely are cause to bias. Women with a prevalent breast cancer at baseline are more likely to decline participation in a prospective study making them subject to selection bias. Moreover, a recent study has shown reduced lactation in these women [16].

The mammography screening program was fully initiated in Malmö 1990 and during 1991 – 1996 the average participation rate was 65% [17,18]. This could have led to over diagnosis of breast cancer, but in our study only incident invasive breast cancers were included in the subgroup analyses. Further tumour classification with regard to the biomarkers, were analyzed using the TMA technique which is a well-documented method for tumour tissue screening and two cores are considered to be sufficient in order to get a representative sample [19,20]. The IHC data was not retrieved in medical records since laboratory analyses were subject to changes over this period of time and hence analyzing all tumours again within TMAs by one pathologist was considered more accurate.

Women in the MDCS were probably selected towards higher socioeconomic groups and the participants in the MDCS had a higher incidence of breast cancer as compared to the rest of the female population in Malmö [6]. However, absolute risks may not be applicable to the background population, but, as we had a large variation in exposure within our sample, we consider that it is still possible to make internal comparisons, thus calculating valid relative risks.

In this study it is important to consider chance findings. There is a risk of a type I error due to the many analyses in this study and our previous study on parity and risk for different breast cancer subgroups was carried out in the same cohort MDCS [21]. No correction for multiple testing was made. As for type II errors, due to few individuals in some analyses, the confidence intervals were wide and the statistical power was relatively low which may have lead to a type II error in some comparisons. And it is important to consider confounding especially in the subgroup analyses, and socioeconomic status might have an impact on breastfeeding pattern. Thulier et al. have reviewed variables associated with breastfeeding and concluded that higher educated and married women, tend to breastfeed their children for longer periods [22]. The relationship between increased risk of unfavourable breast cancer characteristics and time of breastfeeding could be measure of the association between socioeconomic status and breast cancer subgroups. In the MDCS, there was information available on education, type of occupation and marital status/cohabiting and all multivariate analyses were adjusted for these possible confounders. Hence, socioeconomic status should not have affected the results of these analyses. It has previously been shown that women in lower socioeconomic classes have a lower attendance in mammography screening as compared to women in higher socioeconomic classes [17]. This could possibly lead to detection bias, as women in higher socioeconomic classes would more likely have their breast cancer detected earlier; hence more likely have breast cancer with less aggressive characteristics and this would lead to a spurious association between breastfeeding and less aggressive breast cancer tumours. As the results in the present study were in the opposite direction, it makes such an association/detection bias unlikely.

### Previous studies

The breast cancer characteristics Ki67 and grade have to our knowledge not been investigated with regard to breastfeeding previously. Most previous studies on breastfeeding and breast cancer markers, have investigated histological type and hormone receptor status. One previous study found increasing total time of breastfeeding protective against ductal type of breast cancer [5] opposite the non-significant findings in our study. Ursin et al. found total duration of breastfeeding to be protective against ER + PR + and ER-PR- tumours [5], and yet another found breastfeeding for more than six months to be protective against triple-negative breast tumours [4]. These findings were not confirmed in this study.

### Potential explanations

Breastfeeding reduces lifetime ovulatory menstrual cycles [23] i.e. reducing the impact of hormone levels present during normal menstrual cycles [24] and by specifically reducing the progesterone exposure [25]. This may explain the finding in previous studies of a reduced risk of breast cancer in women who had breastfed. It is possible to hypothesize that an environment with relatively low levels of estrogen/progesterone may develop certain kind of tumour sub-groups, i.e. hormone independent tumours which in most cases are prognostically unfavourable tumours. We could in this study there not find any statistically significant association between breastfeeding and ER- tumours. Moreover breastfeeding stimulate the production of prolactin, a hormone that has been reported to have tumour promoting effects [26]. The potential relation between breastfeeding, prolactin and breast cancer is, however complex. Even if prolactin levels are high during lactation, it has been reported that among non-lactating women, prolactin levels in blood are relatively low in women with a previous long duration of breastfeeding [27]. Moreover, breast tissue itself may be able to produce prolactin and this would probably lead to locally increased levels which are not detectable in ordinary blood samples [28]. The potential relationship between breast cancer subgroups and prolactin will however need to be studied in experimental studies, in order to investigate this further.

Generally, factors associated with an increased risk of breast cancer, e.g. HRT [10] and obesity [29] have been associated with prognostically relatively favorable breast tumours. Even if this study did not find any association of breastfeeding and reduced risk of breast cancer, it may be possible to hypothesize that the few associations between breastfeeding duration and aggressive breast cancer characteristics may reflect the same mechanism. It is possible to hypothesize that reproductive factors that are protective against breast cancer may also promote tumours that are non-hormone dependent and hence tumours that grow more autonomous. This should give rise to more aggressive characteristics when studying breast cancer subgroups with regard to reproductive factors as exposures. However, the biological mechanism behind this hypothesis has still to be identified.

## Conclusions

Breastfeeding duration was not associated with breast cancer risk and no strong results were seen with regard to breast cancer subgroups.

### Availability of data

This study was carried out in the Malmö Diet and Cancer Study (MDCS). In order to get access to the data, an application to the committee is needed. Please contact Prof. Jonas Manjer: jonas.manjer@med.lu.se.

## Competing interests

The authors declare that they have no conflict of interests.

## Authors’ contributions

SB participated in designing the study, carried out all the statistical analyses, participated in interpreting the results, reviewed the literature and drafted the manuscript. SiB participated in the study design, analyzing the tumours, interpreting the results and critically revised the manuscript. LA participated in the tumour analyses and critically revised the manuscript. GL participated in the study design, critically revised the results and the manuscript. JM designed the study, supervised and participated in analyzing all statistical analyses and critically revised the manuscript. All authors have read and given approval of the final manuscript.

## Supplementary Material

Additional file 1: Table S1 Distribution of risk factors in different quartiles of average duration of breastfeeding. **Table S2.** Risk of breast cancer subgroups defined by clinico-pathological markers in relation to average duration of breastfeeding. **Table S3.** Risk of breast cancer subgroups defined by immunohistochemical markers in relation to average duration of breastfeeding. **Table S4.** Risk of breast cancer subgroups defined by type and receptor status in relation to average duration of breastfeeding. **Table S5.** Distribution of risk factors in different quartiles of breastfeeding duration of first child. **Table S6.** Risk of breast cancer subgroups defined by clinico-pathological markers in relation to breastfeeding duration of first child. **Table S7.** Risk of breast cancer subgroups defined by immunohistochemical markers in relation to breastfeeding duration of first child. **Table S8.** Risk of breast cancer subgroups defined by type and receptor status in relation to breastfeeding duration of first child. Click here for file
