# Supplementary material for: Breastfeeding in relation to risk of different breast cancer characteristics
Source: BMC Res Notes. 2014 Apr 7;7:216. doi: 10.1186/1756-0500-7-216 (PMC4022388; doi:10.1186/1756-0500-7-216)
Supplement: Additional file 1: Table S1 — Distribution of risk factors in different quartiles of average duration of breastfeeding. Table S2. Risk of breast cancer subgroups defined by clinico-pathological markers in relation to average duration of breastfeeding. Table S3. Risk of breast cancer subgroups defined by immunohistochemical markers in relation to average duration of breastfeeding. Table S4. Risk of breast cancer subgroups defined by type and receptor status in relation to average duration of breastfeeding. Table S5. Distribution of risk factors in different quartiles of breastfeeding duration of first child. Table S6. Risk of breast cancer subgroups defined by clinico-pathological markers in relation to breastfeeding duration of first child. Table S7. Risk of breast cancer subgroups defined by immunohistochemical markers in relation to breastfeeding duration of first child. Table S8. Risk of breast cancer subgroups defined by type and receptor status in relation to breastfeeding duration of first child. [file 1756-0500-7-216-S1.doc]

***Table S1* Distribution of risk factors in different quartiles of average duration of breastfeeding**

| Time in months:  Total: 14092 | <2.2  n = 3398 | >2.2 - <4.0  n = 2988 | >4.0 - <6.2  n = 4160 | >6.2  n = 2985 | Missing  n = 561 |
| --- | --- | --- | --- | --- | --- |
|  | Column percent *(mean and SD in italics)* | | | | |
| Age at baseline (years) |  |  |  |  |  |
| *Mean (SD)* | *55.7 (7.5)* | *56.6 (7.6)* | *57.9 (7.7)* | *58.6 (8.4)* | *57.7 (6.7)* |
| Education (n) |  |  |  |  |  |
| O-level college | 74.1 | 73.9 | 68.4 | 64.7 | 80.6 |
| A-level college | 7.4 | 6.2 | 7.4 | 6.6 | 5.0 |
| University | 18.2 | 19.7 | 24.1 | 28.4 | 13.0 |
| Type of occupation |  |  |  |  |  |
| Manual worker | 42.4 | 42.2 | 36.8 | 37.5 | 45.6 |
| Non-manual worker | 49.2 | 49.7 | 55.3 | 52.3 | 47.1 |
| Employer-self-employed | 7.3 | 7.1 | 7.1 | 8.9 | 5.5 |
| Married/cohabiting |  |  |  |  |  |
| No | 30.6 | 30.3 | 29.0 | 28.7 | 35.5 |
| Yes | 69.4 | 69.7 | 71.0 | 71.2 | 64.2 |
| Age at menarche |  |  |  |  |  |
| <12 | 23.4 | 22.5 | 20.8 | 22.0 | 22.5 |
| >12 to <15 | 53.4 | 53.7 | 54.2 | 51.2 | 47.1 |
| >15 | 22.9 | 23.0 | 24.6 | 26.2 | 27.1 |
| Parity |  |  |  |  |  |
| 1 | 31.9 | 17.2 | 23.8 | 22.4 | 40.1 |
| 2 | 45.4 | 51.2 | 49.9 | 48.0 | 36.5 |
| >3 | 22.7 | 31.6 | 26.4 | 29.5 | 23.4 |
| Age at first childbirth |  |  |  |  |  |
| <20 | 21.0 | 24.4 | 17.8 | 15.6 | 19.4 |
| >20 to <25 | 41.5 | 43.2 | 41.9 | 38.8 | 34.9 |
| >25 to <30 | 26.7 | 25.6 | 30.1 | 30.8 | 28.3 |
| >30 | 10.9 | 6.8 | 10.2 | 14.7 | 15.9 |
| Bilateral oophorectomy |  |  |  |  |  |
| No | 98.7 | 98.3 | 98.7 | 98.6 | 98.8 |
| Yes | 1.3 | 1.7 | 1.3 | 1.4 | 1.2 |
| Age at menopause |  |  |  |  |  |
| Pre-/Perimenopausal | 40.4 | 35.8 | 30.3 | 30.6 | 26.0 |
| <45 | 14.2 | 12.2 | 11.5 | 11.6 | 15.0 |
| >45 to <53 | 33.6 | 37.9 | 40.4 | 39.0 | 43.1 |
| >53 | 10.5 | 12.6 | 16.1 | 17.5 | 12.5 |
| Exposure to OC (ever/never) |  |  |  |  |  |
| No | 44.9 | 45.0 | 50.2 | 55.9 | 52.8 |
| Yes | 55.1 | 55.0 | 49.8 | 44.1 | 46.7 |
| Exposure to HRT* |  |  |  |  |  |
| No | 79.8 | 79.5 | 80.0 | 82.9 | 81.7 |
| ERT | 7.5 | 7.5 | 8.4 | 8.0 | 6.0 |
| PRT | 0.3 | 0.5 | 0.3 | 0.3 | 0.0 |
| CHRT | 11.9 | 12.3 | 10.9 | 8.6 | 12.1 |
| Height |  |  |  |  |  |
| *Mean (SD)* | *163.6 (6.0)* | *163.6 (6.0)* | *163.6 (5.9)* | *163.4 (6.1)* | *162.8 (6.2)* |
| Body mass index |  |  |  |  |  |
| *Mean (SD)* | *25.5 (4.5)* | *25.5 (4.5)* | *25.3 (4.1)* | *25.5 (4.5)* | *26.0 (4.8)* |
| Alcohol consumption |  |  |  |  |  |
| Nothing last year (teetotaler) | 11.5 | 9.6 | 10.1 | 13.2 | 17.5 |
| Something last year (not last month) | 12.1 | 11.7 | 11.3 | 12.7 | 13.7 |
| Something last month | 76.2 | 78.5 | 78.4 | 73.9 | 67.6 |
| Smoking |  |  |  |  |  |
| Never | 37.9 | 38.7 | 46.1 | 52.1 | 44.4 |
| Current | 33.2 | 32.4 | 25.8 | 21.4 | 33.9 |
| Ex | 28.9 | 28.9 | 28.1 | 26.5 | 21.7 |
|  |  |  |  |  |  |

Percentages do not always add up to 100% due to missing. *Current use in peri- and postmenopausal women n = 10288.

***Table S2* Risk of breast cancer subgroups defined by clinico-pathological markers in relation to average duration of breastfeeding**

| Tumour subgroup | Breastfeeding / child in months | Number of Cases | Incidence / 100000 | RR | RR* |
| --- | --- | --- | --- | --- | --- |
|  |  |  |  |  |  |
| Invasive |  |  |  |  |  |
| breast cancer** | <2.2 | 93 | 269 | 1.00 | 1.00 |
|  | >2.2 - <4.0 | 83 | 272 | 1.01 (0.75 - 1.36) | 0.99 (0.73 - 1.33) |
|  | >4.0 - <6.2 | 132 | 309 | 1.15 (0.88 - 1.49) | 1.13 (0.86 - 1.48) |
|  | >6.2 | 92 | 304 | 1.13 (0.85 - 1.51) | 1.15 (0.86 - 1.55) |
|  | Missing | 24 | 399 | --- | --- |
|  | Total | 424 | 294 | *p-trend: 0.27* | *p-trend: 0.19* |
| CIS |  |  |  |  |  |
|  | <2.2 | 9 | 26 | 1.00 | 1.00 |
|  | >2.2 - <4.0 | 13 | 43 | 1.64 (0.70 - 3.83) | 1.87 (0.79 - 4.41) |
|  | >4.0 - <6.2 | 21 | 49 | 1.90 (0.87 - 4.14) | 2.19 (0.99 - 4.83) |
|  | >6.2 | 11 | 36 | 1.40 (0.58 - 3.38) | 1.60 (0.65 - 3.94) |
|  | Missing | 3 | 50 | --- | --- |
|  | Total | 57 | 40 | *p-trend:0.37* | *p-trend:0.20* |
| Size <20mm^#^ |  |  |  |  |  |
|  | <2.2 | 67 | 194 | 1. 00 | 1.00 |
|  | >2.2 - <4.0 | 58 | 190 | 0.98 (0.69 - 1.39) | 0.95 (0.66 - 1.35) |
|  | >4.0 - <6.2 | 93 | 218 | 1.12 (0.82 - 1.53) | 1.08 (0.78 - 1.48) |
|  | >6.2 | 71 | 234 | 1.21 (0.87 - 1.69) | 1.21 (0.86 - 1.71) |
|  | Missing | 15 | 250 | --- | --- |
|  | Total | 304 | 211 | *p-trend:0.19* | *p-trend:0.18* |
| Size >20mm |  |  |  |  |  |
|  | <2.2 | 25 | 72 | 1.00 | 1.00 |
|  | >2.2 - <4.0 | 25 | 82 | 1.13 (0.65 - 1.97) | 1.17 (0.67 - 2.05) |
|  | >4.0 - <6.2 | 38 | 89 | 1.23 (0.74 - 2.03) | 1.30 (0.78 - 2.16) |
|  | >6.2 | 21 | 69 | 0.96 (0.54 - 1.72) | 1.01 (0.56 - 1.83) |
|  | Missing | 9 | 150 | --- | --- |
|  | Total | 118 | 82 | *p-trend:0.94* | *p-trend:0.71* |
| Axillary lymph |  |  |  |  |  |
| node neg^#^ | <2.2 | 27 | 78 | 1.00 | 1.00 |
|  | >2.2 - <4.0 | 32 | 105 | 1.34 (0.80 - 2.24) | 1.35 (0.81 - 2.27) |
|  | >4.0 - <6.2 | 32 | 75 | 0.96 (0.57 - 1.60) | 1.02 (0.61 - 1.72) |
|  | >6.2 | 33 | 109 | 1.40 (0.84 - 2.32) | 1.51 (0.89 - 2.54) |
|  | Missing | 5 | 83 | --- | ---) |
|  | Total | 129 | 89 | *p-trend:0.44* | *p-trend:0.27* |
| Axillary lymph |  |  |  |  |  |
| node pos | <2.2 | 65 | 188 | 1.00 | 1.00 |
|  | >2.2 - <4.0 | 48 | 157 | 0.83 (0.57 - 1.21) | 0.81 (0.56 - 1.19) |
|  | >4.0 - <6.2 | 96 | 225 | 1.19 (0.87 - 1.63) | 1.14 (0.82 - 1.56) |
|  | >6.2 | 58 | 191 | 1.02 (0.72 - 1.45) | 1.01 (0.70 - 1.45) |
|  | Missing | 19 | 316 | --- | --- |
|  | Total | 286 | 198 | *p-trend:0.43* | *p-trend:0.44* |
| Grade I^#^ |  |  |  |  |  |
|  | <2.2 | 31 | 90 | 1.00 | 1.00 |
|  | >2.2 - <4.0 | 24 | 79 | 0.88 (0.51 - 1.49) | 0.84 (0.49 - 1.45) |
|  | >4.0 - <6.2 | 41 | 96 | 1.07 (0.67 - 1.70) | 0.99 (0.61 - 1.59) |
|  | >6.2 | 24 | 79 | 0.89 (0.52 - 1.51) | 0.88 (0.51 - 1.51) |
|  | Missing | 7 | 116 | --- | --- |
|  | Total | 127 | 88 | *p-trend:0.89* | *p-trend:0.88* |
| Grade II |  |  |  |  |  |
|  | <2.2 | 41 | 118 | 1.00 | 1.00 |
|  | >2.2 - <4.0 | 38 | 124 | 1.05 (0.67 - 1.63) | 0.99 (0.63 - 1.55) |
|  | >4.0 - <6.2 | 60 | 140 | 1.18 (0.79 - 1.75) | 1.11 (0.74 - 1.66) |
|  | >6.2 | 40 | 132 | 1.11 (0.72 - 1.72) | 1.04 (0.67 - 1.63) |
|  | Missing | 12 | 199 | --- | --- |
|  | Total | 191 | 132 | *p-trend:0.50* | *p-trend:0.70* |
| Grade III |  |  |  |  |  |
|  | <2.2 | 21 | 61 | 1.00 | 1.00 |
|  | >2.2 - <4.0 | 21 | 69 | 1.13 (0.62 - 2.07) | 1.20 (0.65 - 2.22) |
|  | >4.0 - <6.2 | 30 | 70 | 1.16 (0.66 - 2.02) | 1.33 (0.75 - 2.34) |
|  | >6.2 | 28 | 92 | 1.53 (0.87 - 2.69) | 1.87 (1.05 - 3.34) |
|  | Missing | 5 | 83 | --- | --- |
|  | Total | 105 | 73 | *p-trend:0.16* | *p-trend:0.03* |

* Adjusted for age at baseline (continous), education, socialeconomic status, marrital status, age at menarche, age at first birth, parity, oophorectomy, age at menopause, oral contraceptive use, hormone replacement therapy use, bmi, alcoholconsumption, smoking and height.

** All cases with unilateral invasive breast cancer with tissue samples avaible for examintation. ^#^ Reference group in heterogeneity analyses.

***Table S3* Risk of breast cancer subgroups defined by immunohistochemical markers in relation to average duration of breastfeeding**

| Tumour subgroup | Breastfeeding / child in months | Number of Cases | Incidence / 100000 | RR | RR* |
| --- | --- | --- | --- | --- | --- |
|  |  |  |  |  |  |
| Ki67 low ^#^ |  |  |  |  |  |
| (<10%) | <2.2 | 69 | 199 | 1.00 | 1.00 |
|  | >2.2 - <4.0 | 55 | 180 | 0.90 (0.63 - 1.28) | 0.87 (0.61 - 1.24) |
|  | >4.0 - <6.2 | 82 | 192 | 0.96 (0.70 - 1.32) | 0.91 (0.66 - 1.26) |
|  | >6.2 | 54 | 178 | 0.90 (0.63 - 1.28) | 0.88 (0.61 - 1.27) |
|  | Missing | 11 | 183 | --- | --- |
|  | Total | 271 | 188 | *p-trend:0.64* | *p-trend:0.62* |
| Ki67 high |  |  |  |  |  |
| (>10%) | <2.2 | 16 | 46 | 1.00 | 1.00 |
|  | >2.2 - <4.0 | 17 | 56 | 1.20 (0.61 - 2.38) | 1.24 (0.62 - 2.46) |
|  | >4.0 - <6.2 | 34 | 80 | 1.71 (0.95 - 3.10) | 1.88 (1.03 - 3.43) |
|  | >6.2 | 27 | 89 | 1.93 (1.04 - 3.58) | 2.15 (1.14 - 4.05) |
|  | Missing | 7 | 116 | --- | --- |
|  | Total | 101 | 70 | *p-trend:0.02* | *p-trend:0.005* |
| HER2 ^#^ |  |  |  |  |  |
| (0-1+) | <2.2 | 69 | 199 | 1.00 | 1.00 |
|  | >2.2 - <4.0 | 60 | 196 | 0.98 (0.69 - 1.39) | 0.95 (0.67 - 1.34) |
|  | >4.0 - <6.2 | 98 | 229 | 1.14 (0.84 - 1.55) | 1.11 (0.81 - 1.52) |
|  | >6.2 | 65 | 215 | 1.08 (0.77 - 1.51) | 1.11 (0.79 - 1.57) |
|  | Missing | 19 | 316 | --- | ---) |
|  | Total | 311 | 216 | *p-trend:0.46* | *p-trend:0.30* |
| HER2 |  |  |  |  |  |
| (2+-3+) | <2.2 | 9 | 26 | 1.00 | 1.00 |
|  | >2.2 - <4.0 | 9 | 29 | 1.13 (0.45 - 2.85) | 1.20 (0.47 - 3.07) |
|  | >4.0 - <6.2 | 13 | 30 | 1.18 (0.50 - 2.76) | 1.34 (0.56 - 3.17) |
|  | >6.2 | 9 | 30 | 1.14 (0.45 - 2.87) | 1.22 (0.47 - 3.14) |
|  | Missing | 1 | 17 | --- | --- |
|  | Total | 41 | 28 | *p-trend:0.76* | *p-trend:0.66* |
| Cyclin D1 low^#^ |  |  |  |  |  |
| (<10%) | <2.2 | 67 | 194 | 1.00 | 1.00 |
|  | >2.2 - <4.0 | 62 | 203 | 1.05 (0.74 - 1.48) | 1.01 (0.71 - 1.43) |
|  | >4.0 - <6.2 | 84 | 197 | 1.01 (0.74 - 1.40) | 0.98 (0.71 - 1.36) |
|  | >6.2 | 69 | 228 | 1.18 (0.84 - 1.65) | 1.19 (0.84 - 1.68) |
|  | Missing | 13 | 216 | --- | --- |
|  | Total | 295 | 205 | *p-trend:0.42* | *p-trend:0.34* |
| Cyclin D1 high |  |  |  |  |  |
| (>10%) | <2.2 | 14 | 40 | 1.00 | 1.00 |
|  | >2.2 - <4.0 | 10 | 33 | 0.80 (0.36 - 1.80) | 0.85 (0.37 - 1.92) |
|  | >4.0 - <6.2 | 33 | 77 | 1.88 (1.01 - 3.51) | 1.99 (1.06 - 3.76) |
|  | >6.2 | 15 | 50 | 1.23 (0.59 - 2.54) | 1.29 (0.61 - 2.71) |
|  | Missing | 6 | 100 | --- | --- |
|  | Total | 78 | 54 | *p-trend:0.16* | *p-trend:0.12* |
| P27 low ^#^ |  |  |  |  |  |
| (<10%) | <2.2 | 30 | 87 | 1.00 | 1.00 |
|  | >2.2 - <4.0 | 30 | 98 | 1.13 (0.68 - 1.88) | 1.12 (0.67 - 1.87) |
|  | >4.0 - <6.2 | 29 | 68 | 0.78 (0.47 - 1.31) | 0.81 (0.49 - 1.36) |
|  | >6.2 | 39 | 129 | 1.48 (0.92 - 2.39) | 1.55 (0.95 - 2.54) |
|  | Missing | 7 | 116 | --- | --- |
|  | Total | 135 | 94 | *p-trend:0.28* | *p-trend:0.19* |
| P27 high |  |  |  |  |  |
| (>10%) | <2.2 | 51 | 147 | 1.00 | 1.00 |
|  | >2.2 - <4.0 | 41 | 134 | 0.91 (0.60 - 1.37) | 0.87 (0.58 - 1.32) |
|  | >4.0 - <6.2 | 86 | 201 | 1.36 (0.96 - 1.92) | 1.29 (0.91 - 1.84) |
|  | >6.2 | 41 | 135 | 0.92 (0.61 - 1.39) | 0.92 (0.60 - 1.40) |
|  | Missing | 11 | 183 | --- | --- |
|  | Total | 230 | 159 | *p-trend:0.62* | *p-trend:0.57* |

*Adjusted for age at baseline (continous),, education, socialeconomic status, marrital status, age at menarche, age at first birth, parity, oophorectomy, age at menopause, oral contraceptive use, hormone replacement therapy use, bmi, alcoholconsumption, smoking and height.

^#^Reference group in heterogeneity analyses.

***Table S4 Risk of breast cancer subgroups defined by type and receptor status in relation to average duration of breastfeeding***

| Tumour subgroup | Breastfeeding / child in months | Number of Cases | Incidence / 100000 | RR | RR* |
| --- | --- | --- | --- | --- | --- |
|  |  |  |  |  |  |
| Ductal^#^ |  |  |  |  |  |
|  | <2.2 | 67 | 194 | 1.00 | 1.00 |
|  | >2.2 - <4.0 | 60 | 196 | 1.01 (0.71 - 1.43) | 1.01 (0.71 - 1.43) |
|  | >4.0 - <6.2 | 87 | 204 | 1.05 (0.76 - 1.44) | 1.06 (0.77 - 1.47) |
|  | >6.2 | 72 | 238 | 1.23 (0.88 - 1.71) | 1.31 (0.93 - 1.84) |
|  | Missing | 12 | 199 | --- | --- |
|  | Total | 298 | 207 | *p-trend:0.24* | *p-trend:0.10* |
| Lobular |  |  |  |  |  |
|  | <2.2 | 17 | 49 | 1.00 | 1.00 |
|  | >2.2 - <4.0 | 17 | 56 | 1.13 (0.58 - 2.22) | 1.05 (0.53 - 2.06) |
|  | >4.0 - <6.2 | 26 | 61 | 1.24 (0.67 - 2.28) | 1.10 (0.59 - 2.04) |
|  | >6.2 | 13 | 43 | 0.88 (0.43 - 1.80) | 0.76 (0.37 - 1.59) |
|  | Missing | 9 | 150 | --- | --- |
|  | Total | 82 | 57 | *p-trend:0.89* | *p-trend:0.60* |
| Tubular |  |  |  |  |  |
|  | <2.2 | 7 | 20 | 1.00 | 1.00 |
|  | >2.2 - <4.0 | 4 | 13 | 0.65 (0.19 - 2.21) | 0.65 (0.19 - 2.25) |
|  | >4.0 - <6.2 | 11 | 26 | 1.27 (0.49 - 3.27) | 1.26 (0.48 - 3.32) |
|  | >6.2 | 5 | 12 | 0.82 (0.26 - 2.58) | 0.86 (0.27 - 2.80) |
|  | Missing | 2 | 33 | --- | --- |
|  | Total | 29 | 20 | *p-trend:0.92* | *p-trend:0.83* |
| ERα neg <10%^#^ |  |  |  |  |  |
|  | <2.2 | 10 | 29 | 1.00 | 1.00 |
|  | >2.2 - <4.0 | 12 | 39 | 1.34 (0.58 - 3.11) | 1.39 (0.59 - 3.25) |
|  | >4.0 - <6.2 | 13 | 30 | 1.04 (0.46 - 2.38) | 1.08 (0.47 - 2.50) |
|  | >6.2 | 14 | 46 | 1.60 (0.71 - 3.60) | 1.62 (0.70 - 3.74) |
|  | Missing | 1 | 17 | --- | --- |
|  | Total | 50 | 35 | *p-trend:0.34* | *p-trend:0.38* |
| ERα pos >10% |  |  |  |  |  |
|  | <2.2 | 74 | 214 | 1.00 | 1.00 |
|  | >2.2 - <4.0 | 59 | 193 | 0.90 (0.64 - 1.27) | 0.88 (0.62 - 1.24) |
|  | >4.0 - <6.2 | 109 | 255 | 1.19 (0.88 - 1.60) | 1.16 (0.86 - 1.56) |
|  | >6.2 | 72 | 238 | 1.11 (0.81 - 1.54) | 1.13 (0.81 - 1.57) |
|  | Missing | 20 | 332 | --- | --- |
|  | Total | 334 | 232 | *p-trend:0.24* | *p-trend:0.18* |
| ERβ neg <10%^#^ |  |  |  |  |  |
|  | <2.2 | 36 | 104 | 1.00 | 1.00 |
|  | >2.2 - <4.0 | 31 | 101 | 0.98 (0.60 - 1.58) | 0.95 (0.59 - 1.55) |
|  | >4.0 - <6.2 | 47 | 110 | 1.06 (0.69 - 1.63) | 1.07 (0.69 - 1.67) |
|  | >6.2 | 37 | 122 | 1.17 (0.74 - 1.86) | 1.23 (0.77 - 1.97) |
|  | Missing | 9 | 150 | --- | --- |
|  | Total | 160 | 111 | *p-trend:0.46* | *p-trend:0.30* |
| ERβ pos >10% |  |  |  |  |  |
|  | <2.2 | 34 | 98 | 1.00 | 1.00 |
|  | >2.2 - <4.0 | 33 | 108 | 1.09 (0.67 - 1.75) | 1.08 (0.67 - 1.75) |
|  | >4.0 - <6.2 | 53 | 124 | 1.24 (0.81 - 1.91) | 1.22 (0.79 - 1.89) |
|  | >6.2 | 27 | 89 | 0.91 (0.55 - 1.51) | 0.93 (0.56 - 1.56) |
|  | Missing | 9 | 150 | --- | --- |
|  | Total | 156 | 108 | *p-trend:0.99* | *p-trend:0.90* |
| PgR neg <10%^#^ |  |  |  |  |  |
|  | <2.2 | 47 | 136 | 1.00 | 1.00 |
|  | >2.2 - <4.0 | 44 | 144 | 1.06 (0.70 - 1.60) | 1.04 (0.69 - 1.58) |
|  | >4.0 - <6.2 | 58 | 136 | 1.00 (0.68 - 1.46) | 1.00 (0.67 - 1.47) |
|  | >6.2 | 41 | 135 | 1.00 (0.66 - 1.52) | 1.06 (0.69 - 1.63) |
|  | Missing | 9 | 150 | --- | --- |
|  | Total | 199 | 138 | *p-trend:0.92* | *p-trend:0.80* |
| PgR pos >10% |  |  |  |  |  |
|  | <2.2 | 33 | 95 | 1.00 | 1.00 |
|  | >2.2 - <4.0 | 25 | 82 | 0.85 (0.51 - 1.43) | 0.85 (0.50 - 1.43) |
|  | >4.0 - <6.2 | 55 | 129 | 1.34 (0.87 - 2.06) | 1.31 (0.85 - 2.03) |
|  | >6.2 | 36 | 119 | 1.25 (0.78 - 2.00) | 1.22 (0.75 - 1.98) |
|  | Missing | 10 | 166 | --- | --- |
|  | Total | 159 | 110 | *p-trend:0.14* | *p-trend:0.15* |

* Adjusted for age at baseline (continous),, education, socialeconomic status, marrital status, age at menarche, age at first birth, parity, oophorectomy, age at menopause, oral contraceptive use, hormone replacement therapy use, bmi, alcoholconsumption, smoking and height.

^#^Reference group in heterogeneity analyses.

***Table S5* Distribution of risk factors in different quartiles of breastfeeding duration of first child**

| Time in months:  Total: 14092 | <3.0  n = 3900 | >3.0 - <4.0  n = 2389 | >4.0 - <7.0  n = 4105 | >7.0  n = 2967 | Missing  n = 731 |
| --- | --- | --- | --- | --- | --- |
|  | Column percent *(mean and SD in italics)* | | | |  |
| Age at baseline (years) |  |  |  |  |  |
| *Mean (SD)* | *55.9 (7.7)* | *56.3 (7.4)* | *57.7 (7.8)* | *59.0 (8.1)* | *57.5 (6.8)* |
| Education (n) |  |  |  |  |  |
| O-level college | 74.0 | 73.0 | 67.6 | 66.5 | 78.5 |
| A-level college | 7.5 | 6.2 | 7.4 | 6.3 | 5.3 |
| University | 18.3 | 20.6 | 24.8 | 27.0 | 14.9 |
| Type of occupation |  |  |  |  |  |
| Manual worker | 42.4 | 42.6 | 36.5 | 37.4 | 45.1 |
| Non-manual worker | 49.4 | 49.1 | 55.4 | 52.5 | 47.9 |
| Employer-self-employed | 7.3 | 7.2 | 7.4 | 8.6 | 5.3 |
| Married/cohabiting |  |  |  |  |  |
| No | 30.4 | 29.2 | 29.4 | 29.4 | 34.2 |
| Yes | 69.6 | 70.8 | 70.6 | 70.6 | 65.5 |
| Age at menarche |  |  |  |  |  |
| <12 | 22.6 | 23.3 | 21.0 | 22.0 | 22.0 |
| >12 to <15 | 53.8 | 52.9 | 54.0 | 51.7 | 48.6 |
| >15 | 23.3 | 23.1 | 24.5 | 25.7 | 26.7 |
| Parity |  |  |  |  |  |
| 1 | 27.8 | 21.5 | 24.0 | 22.6 | 30.8 |
| 2 | 46.1 | 51.7 | 49.8 | 48.3 | 37.8 |
| >3 | 26.2 | 26.7 | 26.1 | 29.1 | 31.5 |
| Age at first childbirth |  |  |  |  |  |
| <20 | 22.7 | 23.6 | 16.8 | 15.9 | 20.1 |
| >20 to <25 | 42.0 | 43.0 | 41.7 | 38.7 | 36.9 |
| >25 to <30 | 25.4 | 25.4 | 31.7 | 30.6 | 27.1 |
| >30 | 10.0 | 8.0 | 9.8 | 14.8 | 14.6 |
| Bilateral oophorectomy |  |  |  |  |  |
| No | 98.7 | 98.2 | 98.6 | 98.6 | 98.8 |
| Yes | 1.3 | 1.8 | 1.4 | 1.4 | 1.2 |
| Age at menopause |  |  |  |  |  |
| Pre-/Perimenopausal | 39.9 | 37.1 | 31.9 | 27.5 | 26.8 |
| <45 | 13.9 | 12.6 | 10.9 | 11.7 | 15.6 |
| >45 to <53 | 34.1 | 36.5 | 40.1 | 40.9 | 41.2 |
| >53 | 10.7 | 12.3 | 15.4 | 18.5 | 13.5 |
| Exposure to OC (ever/never) |  |  |  |  |  |
| No | 45.5 | 45.0 | 49.4 | 56.2 | 52.0 |
| Yes | 54.5 | 55.0 | 50.6 | 43.8 | 47.6 |
| Exposure to HRT* |  |  |  |  |  |
| No | 80.8 | 78.8 | 80.0 | 82.1 | 81.0 |
| ERT | 7.4 | 8.1 | 8.1 | 8.0 | 6.7 |
| PRT | 0.3 | 0.5 | 0.2 | 0.3 | 0.2 |
| CHRT | 11.1 | 12.1 | 11.3 | 9.4 | 11.9 |
| Height |  |  |  |  |  |
| *Mean (SD)* | *163.5 (6.0)* | *163.7 (6.0)* | *163.7 (6.0)* | *163.5 (6.0)* | *162.8 (6.3)* |
| Body mass index |  |  |  |  |  |
| *Mean (SD)* | *25.6 (4.5)* | *25.3 (4.4)* | *25.3 (4.2)* | *25.5 (4.4)* | *25.8 (4.8)* |
| Alcohol consumption |  |  |  |  |  |
| Nothing last year (teetotaler) | 11.7 | 9.4 | 9.6 | 13.4 | 16.1 |
| Something last year (not last month) | 11.8 | 11.6 | 11.5 | 12.7 | 13.4 |
| Something last month | 76.3 | 78.9 | 78.7 | 73.6 | 69.5 |
| Smoking |  |  |  |  |  |
| Never | 37.9 | 39.0 | 45.7 | 52.3 | 44.3 |
| Current | 32.8 | 31.7 | 26.3 | 21.5 | 33.1 |
| Ex | 29.2 | 29.2 | 28.0 | 26.2 | 22.6 |
|  |  |  |  |  |  |

Percentages do not always add up to 100% due to missing. *Current in peri- and postmenopausal women n = 10288.

***Table S6* Risk of breast cancer subgroups defined by clinico-pathological markers in relation to breastfeeding duration of first child**

| Tumour subgroup | Breastfeeding first child in months | Number of Cases | Incidence / 100000 | RR | RR* |
| --- | --- | --- | --- | --- | --- |
|  |  |  |  |  |  |
| Invasive |  |  |  |  |  |
| breast cancer** | <3.0 | 108 | 273 | 1.00 | 1.00 |
|  | >3.0 - <4.0 | 56 | 229 | 0.83 (0.60 - 1.15) | 0.82 (0.59 - 1.13) |
|  | >4.0 - <7.0 | 136 | 324 | 1.18 (0.92 - 1.52) | 1.16 (0.89 - 1.49) |
|  | >7.0 | 96 | 316 | 1.15 (0.88 - 1.52) | 1.15 (0.87 - 1.52) |
|  | Missing | 28 | 354 | --- | --- |
|  | Total | 424 | 294 | *p-trend:0.11* | *p-trend:0.10* |
| CIS |  |  |  |  |  |
|  | <3.0 | 13 | 33 | 1.00 | 1.00 |
|  | >3.0 - <4.0 | 12 | 49 | 1.50 (0.68 - 3.28) | 1.55 (0.70 - 3.42) |
|  | >4.0 - <7.0 | 18 | 43 | 1.31 (0.64 - 2.67) | 1.44 (0.70 - 2.96) |
|  | >7.0 | 11 | 36 | 1.11 (0.50 - 2.47) | 1.19 (0.52 - 2.71) |
|  | Missing | 3 | 38 | ---) | --- |
|  | Total | 57 | 40 | *p-trend:0.80* | *p-trend:0.57* |
| Size <20mm^#^ |  |  |  |  |  |
|  | <3.0 | 76 | 192 | 1. 00 | 1.00 |
|  | >3.0 - <4.0 | 41 | 168 | 0.87 (0.59 - 1.27) | 0.85 (0.58 - 1.24) |
|  | >4.0 - <7.0 | 94 | 224 | 1.16 (0.86 - 1.57) | 1.11 (0.82 - 1.51) |
|  | >7.0 | 75 | 247 | 1.28 (0.93 - 1.76) | 1.26 (0.91 - 1.74) |
|  | Missing | 18 | 228 | --- | --- |
|  | Total | 304 | 211 | *p-trend:0.07* | *p-trend:0.08* |
| Size >20mm |  |  |  |  |  |
|  | <3.0 | 31 | 78 | 1.00 | 1.00 |
|  | >3.0 - <4.0 | 15 | 61 | 0.78 (0.42 - 1.44) | 0.77 (0.42 - 1.44) |
|  | >4.0 - <7.0 | 41 | 98 | 1.24 (0.78 - 1.98) | 1.29 (0.80 - 2.07) |
|  | >7.0 | 21 | 69 | 0.88 (0.51 - 1.53) | 0.90 (0.51 - 1.59) |
|  | Missing | 10 | 127 | --- | --- |
|  | Total | 118 | 82 | *p-trend:0.88* | *p-trend:0.71* |
| Axillary lymph |  |  |  |  |  |
| node neg^#^ | <3.0 | 34 | 86 | 1.00 | 1.00 |
|  | >3.0 - <4.0 | 17 | 69 | 0.81 (0.45 - 1.44) | 0.81 (0.45 - 1.45) |
|  | >4.0 - <7.0 | 45 | 107 | 1.24 (0.80 - 1.94) | 1.32 (0.84 - 2.08) |
|  | >7.0 | 28 | 92 | 1.07 (0.65 - 1.77) | 1.14 (0.69 - 1.91) |
|  | Missing | 5 | 63 | --- | --- |
|  | Total | 129 | 89 | *p-trend:0.45* | *p-trend:0.30* |
| Axillary lymph |  |  |  |  |  |
| node pos | <3.0 | 72 | 182 | 1.00 | 1.00 |
|  | >3.0 - <4.0 | 36 | 147 | 0.80 (0.54 - 1.20) | 0.78 (0.52 - 1.17) |
|  | >4.0 - <7.0 | 88 | 210 | 1.15 (0.84 - 1.56) | 1.08 (0.79 - 1.48) |
|  | >7.0 | 67 | 221 | 1.21 (0.86 - 1.68) | 1.16 (0.82 - 1.63) |
|  | Missing | 23 | 291 | --- | --- |
|  | Total | 286 | 198 | *p-trend:0.13* | *p-trend:0.18* |
| Grade I^#^ |  |  |  |  |  |
|  | <3.0 | 35 | 89 | 1.00 | 1.00 |
|  | >3.0 - <4.0 | 18 | 74 | 0.83 (0.47 - 1.46) | 0.79 (0.45 - 1.40) |
|  | >4.0 - <7.0 | 37 | 88 | 0.99 (0.62 - 1.57) | 0.90 (0.56 - 1.44) |
|  | >7.0 | 29 | 96 | 1.08 (0.66 - 1.76) | 1.02 (0.62 - 1.70) |
|  | Missing | 8 | 101 | --- | --- |
|  | Total | 127 | 88 | *p-trend:0.70* | *p-trend:0.85* |
| Grade II |  |  |  |  |  |
|  | <3.0 | 48 | 121 | 1.00 | 1.00 |
|  | >3.0 - <4.0 | 19 | 78 | 0.64 (0.37 - 1.08) | 0.63 (0.37 - 1.07) |
|  | >4.0 - <7.0 | 73 | 174 | 1.42 (0.99 - 2.05) | 1.36 (0.94 - 1.96) |
|  | >7.0 | 37 | 122 | 1.00 (0.65 - 1.53) | 0.93 (0.60 - 1.44) |
|  | Missing | 14 | 177 | --- | --- |
|  | Total | 191 | 132 | *p-trend:0.27* | *p-trend:0.43* |
| Grade III |  |  |  |  |  |
|  | <3.0 | 25 | 63 | 1.00 | 1.00 |
|  | >3.0 - <4.0 | 19 | 78 | 1.22 (0.67 - 2.22) | 1.25 (0.68 - 2.27) |
|  | >4.0 - <7.0 | 25 | 60 | 0.94 (0.54 - 1.64) | 1.05 (0.60 - 1.85) |
|  | >7.0 | 30 | 99 | 1.56 (0.92 - 2.65) | 1.84 (1.07 - 3.18) |
|  | Missing | 6 | 76 | 1--- | --- |
|  | Total | 105 | 73 | *p-trend:0.21* | *p-trend:0.052* |

* Adjusted for age at baseline (continous), education, socialeconomic status, marrital status, age at menarche, age at first birth, parity, oophorectomy, age at menopause, oral contraceptive use, hormone replacement therapy use, bmi, alcoholconsumption, smoking and height.

** All cases with unilateral invasive breast cancer with tissue samples avaible for examintation. ^#^ Reference group in heterogeneity analyses.

***Table S7* Risk of breast cancer subgroups defined by immunohistochemical markers in relation to breastfeeding duration of first child**

| Tumour subgroup | Breastfeeding first child in months | Number of Cases | Incidence / 100000 | RR | RR* |
| --- | --- | --- | --- | --- | --- |
|  |  |  |  |  |  |
| Ki67 low |  |  |  |  |  |
| (<10%) ^#^ | <3.0 | 79 | 200 | 1.00 | 1.00 |
|  | >3.0 - <4.0 | 34 | 139 | 0.69 (0.46 - 1.03) | 0.67 (0.45 - 1.00) |
|  | >4.0 - <7.0 | 92 | 219 | 1.09 (0.81 - 1.47) | 1.03 (0.76 - 1.40) |
|  | >7.0 | 54 | 178 | 0.88 (0.63 - 1.25) | 0.85 (0.60 - 1.21) |
|  | Missing | 12 | 152 | --- | --- |
|  | Total | 271 | 188 | *p-trend:0.99* | *p-trend:0.87* |
| Ki67 high |  |  |  |  |  |
| (>10%) | <3.0 | 20 | 51 | 1.00 | 1.00 |
|  | >3.0 - <4.0 | 13 | 53 | 1.05 (0.52 - 2.10) | 1.06 (0.52 - 2.13) |
|  | >4.0 - <7.0 | 26 | 62 | 1.22 (0.68 - 2.18) | 1.32 (0.73 - 2.38) |
|  | >7.0 | 34 | 112 | 2.20 (1.27 - 3.83) | 2.44 (1.38 - 4.29) ^##^ |
|  | Missing | 8 | 101 | --- | --- |
|  | Total | 101 | 70 | *p-trend:0.005* | *p-trend:0.001* |
| HER2 (0-1+)^#^ |  |  |  |  |  |
|  | <3.0 | 84 | 213 | 1.00 | 1.00 |
|  | >3.0 - <4.0 | 35 | 143 | 0.67 (0.45 - 0.99) | 0.64 (0.43 - 0.96) |
|  | >4.0 - <7.0 | 102 | 243 | 1.13 (0.85 - 1.51) | 1.10 (0.82 - 1.48) |
|  | >7.0 | 69 | 227 | 1.06 (0.77 - 1.46) | 1.07 (0.77 - 1.48) |
|  | Missing | 21 | 266 | --- | --- |
|  | Total | 311 | 216 | *p-trend:0.29* | *p-trend:0.22* |
| HER2 (2+-3+) |  |  |  |  |  |
|  | <3.0 | 9 | 23 | 1.00 | 1.00 |
|  | >3.0 - <4.0 | 8 | 33 | 1.43 (0.55 - 3.71) | 1.47 (0.56 - 3.83) |
|  | >4.0 - <7.0 | 11 | 26 | 1.16 (0.48 - 2.80) | 1.26 (0.52 - 3.07) |
|  | >7.0 | 11 | 36 | 1.59 (0.66 - 3.84) | 1.71 (0.70 - 4.21) |
|  | Missing | 2 | 25 | --- | --- |
|  | Total | 41 | 28 | *p-trend:0.40* | *p-trend:0.33* |
| Cyclin D1 low |  |  |  |  |  |
| (<10%) ^#^ | <3.0 | 80 | 202 | 1.00 | 1.00 |
|  | >3.0 - <4.0 | 39 | 159 | 0.79 (0.54 - 1.15) | 0.76 (0.52 - 1.12) |
|  | >4.0 - <7.0 | 91 | 217 | 1.07 (0.79 - 1.44) | 1.03 (0.76 - 1.40) |
|  | >7.0 | 70 | 231 | 1.14 (0.82 - 1.57) | 1.13 (0.81 - 1.56) |
|  | Missing | 15 | 190 | --- | --- |
|  | Total | 295 | 205 | *p-trend:0.27* | *p-trend:0.26* |
| Cyclin D1 high |  |  |  |  |  |
| (>10%) | <3.0 | 16 | 40 | 1.00 | 1.00 |
|  | >3.0 - <4.0 | 7 | 29 | 0.69 (0.29 - 1.68) | 0.72 (0.30 - 1.77) |
|  | >4.0 - <7.0 | 27 | 64 | 1.56 (0.84 - 2.89) | 1.65 (0.88 - 3.08) |
|  | >7.0 | 21 | 69 | 1.68 (0.88 - 3.22) | 1.73 (0.89 - 3.36) |
|  | Missing | 7 | 89 | --- | --- |
|  | Total | 78 | 54 | *p-trend:0.04* | *p-trend:0.03* |
| P27 low |  |  |  |  |  |
| (<10%) ^#^ | <3.0 | 36 | 91 | 1.00 | 1.00 |
|  | >3.0 - <4.0 | 17 | 69 | 0.76 (0.43 - 1.36) | 0.77 (0.43 - 1.37) |
|  | >4.0 - <7.0 | 32 | 76 | 0.84 (0.52 - 1.35) | 0.86 (0.53 - 1.39) |
|  | >7.0 | 40 | 132 | 1.45 (0.92 - 2.27) | 1.50 (0.94 - 2.38) |
|  | Missing | 10 | 127 | --- | --- |
|  | Total | 135 | 94 | *p-trend:0.15* | *p-trend:0.10* |
| P27 high |  |  |  |  |  |
| (>10%) | <3.0 | 60 | 152 | 1.00 | 1.00 |
|  | >3.0 - <4.0 | 27 | 110 | 0.72 (0.46 - 1.13) | 0.70 (0.44 - 1.10) |
|  | >4.0 - <7.0 | 85 | 203 | 1.32 (0.95 - 1.84) | 1.26 (0.90 - 1.77) |
|  | >7.0 | 47 | 155 | 1.01 (0.69 - 1.48) | 0.99 (0.67 - 1.46) |
|  | Missing | 11 | 139 | --- | --- |
|  | Total | 230 | 159 | *p-trend:0.32* | *p-trend:0.36* |

* Adjusted for age at baseline (continous), education, socialeconomic status, marrital status, age at menarche, age at first birth, parity, oophorectomy, age at menopause, oral contraceptive use, hormone replacement therapy use, bmi, alcoholconsumption, smoking and height.

^#^ Reference group in heterogeneity analyses. ^##^ Heterogeneity: p = 0.006

***Table S8* Risk of breast cancer subgroups defined by type and receptor status in relation to breastfeeding duration of first child**

| Tumour subgroup | Breastfeeding first child in months | Number of Cases | Incidence / 100000 | RR | RR* |
| --- | --- | --- | --- | --- | --- |
|  |  |  |  |  |  |
| Ductal^#^ |  |  |  |  |  |
|  | <3.0 | 80 | 202 | 1.00 | 1.00 |
|  | >3.0 - <4.0 | 37 | 151 | 0.74 (0.50 - 1.10) | 0.74 (0.50 - 1.09) |
|  | >4.0 - <7.0 | 94 | 224 | 1.10 (0.82 - 1.48) | 1.11 (0.82 - 1.50) |
|  | >7.0 | 72 | 237 | 1.16 (0.85 - 1.60) | 1.22 (0.88 - 1.69) |
|  | Missing | 15 | 190 | --- | --- |
|  | Total | 298 | 207 | *p-trend:0.17* | *p-trend:0.08* |
| Lobular |  |  |  |  |  |
|  | <3.0 | 19 | 48 | 1.00 | 1.00 |
|  | >3.0 - <4.0 | 11 | 45 | 0.94 (0.45 - 1.97) | 0.87 (0.41 - 1.83) |
|  | >4.0 - <7.0 | 27 | 64 | 1.34 (0.74 - 2.41) | 1.16 (0.64 - 2.11) |
|  | >7.0 | 16 | 53 | 1.10 (0.56 - 2.13) | 0.92 (0.47 - 1.81) |
|  | Missing | 9 | 114 | --- | --- |
|  | Total | 82 | 57 | *p-trend:0.52* | *p-trend:0.94* |
| Tubular |  |  |  |  |  |
|  | <3.0 | 7 | 18 | 1.00 | 1.00 |
|  | >3.0 - <4.0 | 7 | 29 | 1.61 (0.56 - 4.58) | 1.59 (0.55 - 4.57) |
|  | >4.0 - <7.0 | 7 | 17 | 0.94 (0.33 - 2.67) | 0.92 (0.32 - 2.65) |
|  | >7.0 | 6 | 20 | 1.11 (0.37 - 3.30) | 1.13 (0.37 - 3.44) |
|  | Missing | 2 | 25 | --- | --- |
|  | Total | 29 | 20 | *p-trend:0.92* | *p-trend:0.96* |
| ERα neg <10%^#^ |  |  |  |  |  |
|  | <3.0 | 13 | 33 | 1.00 | 1.00 |
|  | >3.0 - <4.0 | 7 | 29 | 0.85 (0.34 - 2.13) | 0.85 (0.34 - 2.14) |
|  | >4.0 - <7.0 | 12 | 29 | 0.86 (0.39 - 1.88) | 0.87 (0.39 - 1.93) |
|  | >7.0 | 16 | 53 | 1.58 (0.76 - 3.28) | 1.59 (0.75 - 3.38) |
|  | Missing | 2 | 25 | --- | --- |
|  | Total | 50 | 35 | *p-trend:0.28* | *p-trend:0.26* |
| ERα pos >10% |  |  |  |  |  |
|  | <3.0 | 86 | 218 | 1.00 | 1.00 |
|  | >3.0 - <4.0 | 38 | 155 | 0.71 (0.49 - 1.04) | 0.70 (0.47 - 1.02) |
|  | >4.0 - <7.0 | 113 | 269 | 1.23 (0.93 - 1.63) | 1.19 (0.90 - 1.59) |
|  | >7.0 | 75 | 949 | 1.13 (0.83 - 1.54) | 1.12 (0.82 - 1.54) |
|  | Missing | 22 | 278 | --- | --- |
|  | Total | 334 | 232 | *p-trend:0.11* | *p-trend:0.11* |
| ERβ neg <10%^#^ |  |  |  |  |  |
|  | <3.0 | 42 | 106 | 1.00 | 1.00 |
|  | >3.0 - <4.0 | 20 | 82 | 0.77 (0.45 - 1.31) | 0.75 (0.44 - 1.28) |
|  | >4.0 - <7.0 | 48 | 114 | 1.08 (0.71 - 1.63) | 1.10 (0.72 - 1.67) |
|  | >7.0 | 40 | 132 | 1.24 (0.80 - 1.91) | 1.28 (0.82 - 2.00) |
|  | Missing | 10 | 127 | --- | --- |
|  | Total | 160 | 111 | *p-trend:0.23* | *p-trend:0.15* |
| ERβ pos >10% |  |  |  |  |  |
|  | <3.0 | 43 | 109 | 1.00 | 1.00 |
|  | >3.0 - <4.0 | 22 | 90 | 0.81 (0.48 - 1.35) | 0.80 (0.48 - 1.34) |
|  | >4.0 - <7.0 | 49 | 117 | 1.05 (0.70 - 1.58) | 1.02 (0.67 - 1.55) |
|  | >7.0 | 32 | 105 | 0.95 (0.60 - 1.50) | 0.95 (0.60 - 1.52) |
|  | Missing | 10 | 127 | --- | --- |
|  | Total | 156 | 108 | *p-trend:0.93* | *p-trend:0.90* |
| PgR neg <10%^#^ |  |  |  |  |  |
|  | <3.0 | 56 | 142 | 1.00 | 1.00 |
|  | >3.0 - <4.0 | 31 | 127 | 0.89 (0.57 - 1.38) | 0.87 (0.56 - 1.35) |
|  | >4.0 - <7.0 | 59 | 141 | 0.99 (0.69 - 1.42) | 0.98 (0.68 - 1.42) |
|  | >7.0 | 44 | 145 | 1.02 (0.69 - 1.51) | 1.06 (0.70 - 1.58) |
|  | Missing | 9 | 114 | --- | --- |
|  | Total | 199 | 138 | *p-trend:0.87* | *p-trend:0.71* |
| PgR pos >10% |  |  |  |  |  |
|  | <3.0 | 41 | 104 | 1.00 | 1.00 |
|  | >3.0 - <4.0 | 12 | 49 | 0.47 (0.25 - 0.89) | 0.47 (0.25 - 0.89) |
|  | >4.0 - <7.0 | 56 | 133 | 1.27 (0.85 - 1.91) | 1.25 (0.83 - 1.88) |
|  | >7.0 | 39 | 128 | 1.23 (0.79 - 1.90) | 1.19 (0.76 - 1.86) |
|  | Missing | 11 | 139 | --- | --- |
|  | Total | 159 | 110 | *p-trend:0.08* | *p-trend:0.10* |

* Adjusted for age at baseline (continous), education, socialeconomic status, marrital status, age at menarche, age at first birth, parity, oophorectomy, age at menopause, oral contraceptive use, hormone replacement therapy use, bmi, alcoholconsumption, smoking and height.

^#^ Reference group in heterogeneity analyses.
